# Supplementary material for: IL-22-Activated MUC13 Impacts on Colonic Barrier Function through JAK1/STAT3, SNAI1/ZEB1 and ROCK2/MAPK Signaling
Source: Cells. 2023 Apr 23;12(9):1224. doi: 10.3390/cells12091224 (PMC10177587; doi:10.3390/cells12091224)
Supplement: Supplementary file 1 [file cells-12-01224-s001.zip › cells-2275517-supplementary.pdf]

Supplementary Material

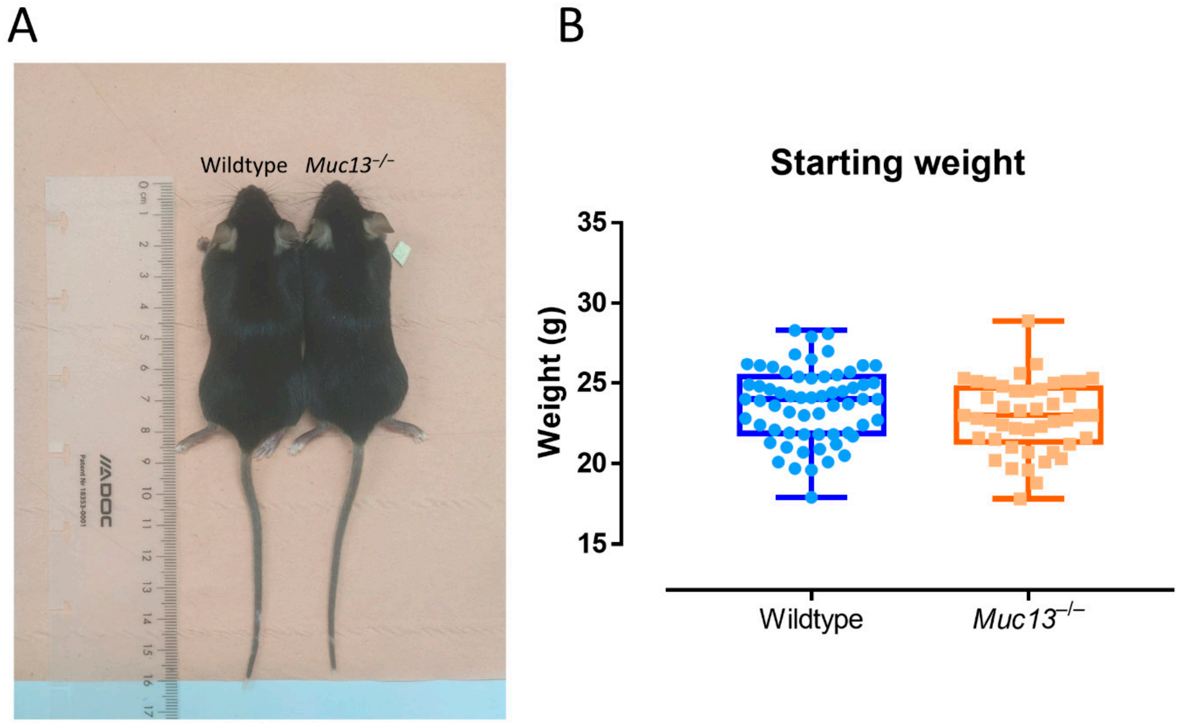

Figure S1. Comparison of healthy wildtype and *Muc13*$^{-/-}$ mice. **(A)** Representative photo of 7-week old male wildtype and *Muc13*$^{-/-}$ mice. **(B)** Weight of wildtype and *Muc13*$^{-/-}$ mice at the start of the DSS experiments.

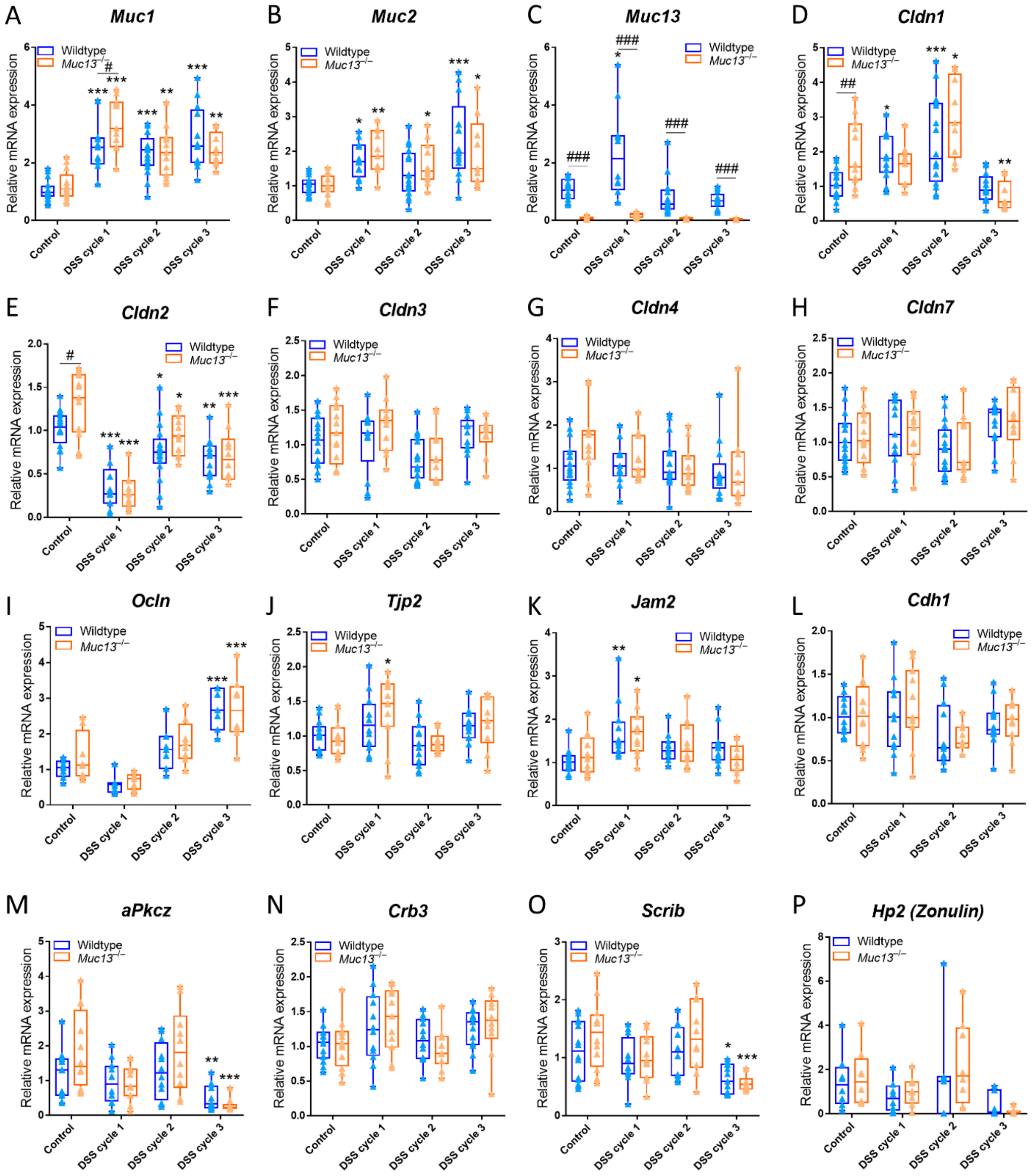

Figure S2. Gene expression of mucins, intercellular junctions and cell polarity complexes in the colon of wildtype and *Muc13*−/− mice during the course of DSS-colitis. Relative mRNA expression of (A-C) mucins (*Muc1*, *Muc2*, *Muc13*), (D-K) tight junctions (*Cldn1*, *Cldn2*, *Cldn3*, *Cldn4*, *Cldn7*, *Ocln*, *Tjp2*, *Jam2*), (L) adherens junctions (*Cdh1*), and (M-O) cell polarity subunits (*Scrib*, *Crb3*, *aPKcz*) and *Hp2* (Zonulin) in the colon of healthy and DSS-colitis wildtype and *Muc13*−/− mice (N=7-17/group/gene). Significant differences between control and colitis mice are indicated by *p<0.05; **p<0.01; ***p<0.001 and between wildtype (WT) and *Muc13*−/− by #p<0.05; ##p<0.01; ###p<0.001 (Two-Way ANOVA).

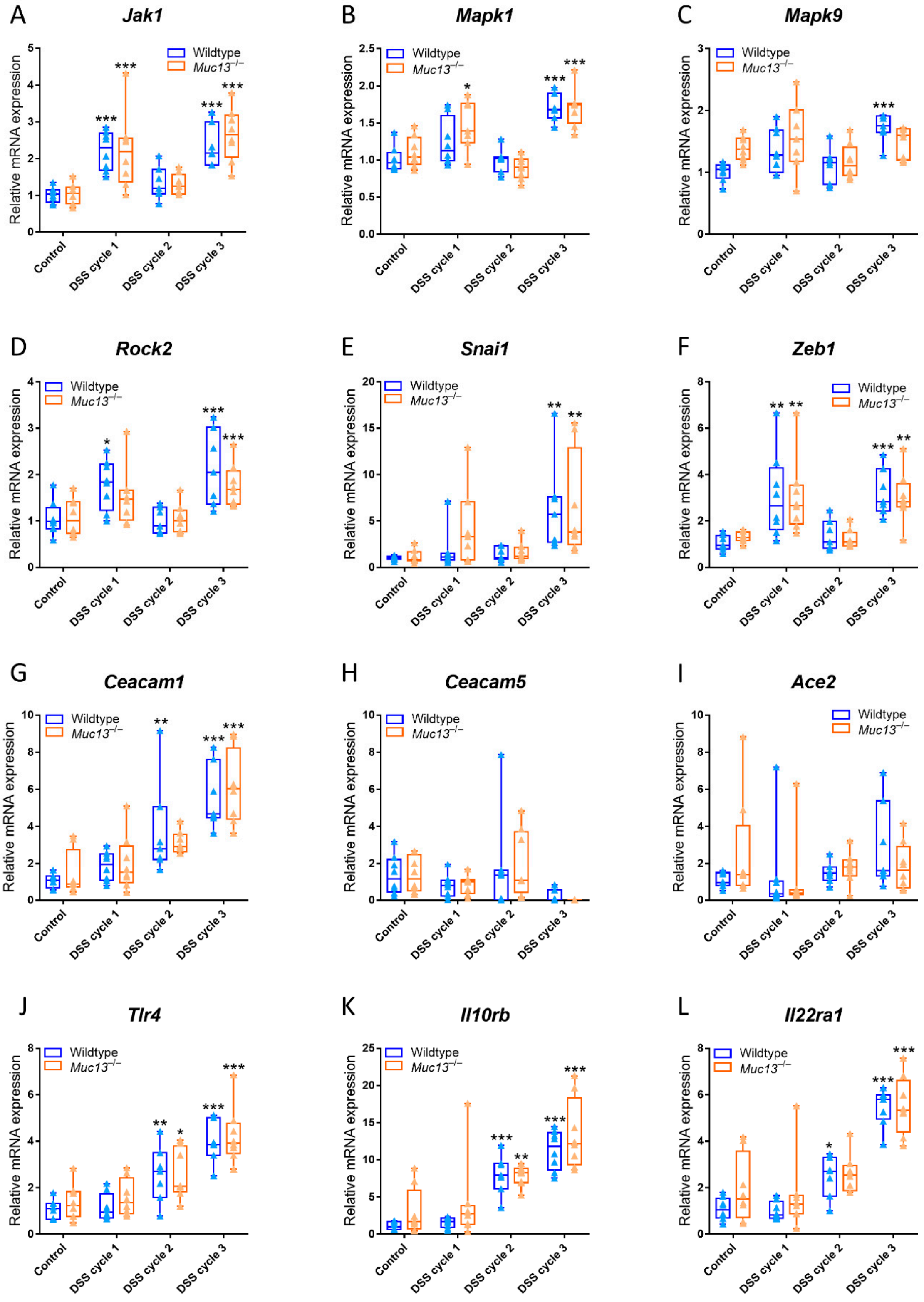

Figure S3. mRNA expression analysis of major regulators and epithelial immunity mediators involved in intestinal barrier homeostasis in the colon of wildtype and *Muc13*−/− mice during the course of DSS-colitis. Relative mRNA expression of (**A**–**F**) major regulators (*Jak1*, *Mapk1*, *Mapk9*, *Rock2*, *Snai1*, *Zeb1*) and (G-L) epithelial immunity mediators (*Ceacam1*, *Ceacam5*, *Ace2*, *Tlr4*, *Il10rb*, *Il22ra*) (N=7-17/group/gene). Significant differences between control and colitis mice are indicated by *p<0.05; **p<0.01; ***p<0.001 (Two-Way ANOVA).

Table S1. Primer sequences used in qPCR assays.

Gene name	Species	Primer	Primer sequence (5′–3′)
ACE2	Human	Hs_ACE2_1_SG QuantiTect Primer Assay	QT00034055
ACTB	Human	Hs_ACTB_1_SG QuantiTect Primer Assay	QT00095431
CEACAM1	Human	Hs_CEACAM1_1_SG QuantiTect Primer Assay	QT00072429
CEACAM5	Human	Hs_CEACAM5_1_SG QuantiTect Primer Assay	QT00032991
CEACAM6	Human	Hs_CEACAM6_1_SG QuantiTect Primer Assay	QT00066521
CDH1	Human	Hs_CDH1_1_SG QuantiTect Primer Assay	QT00080143
CLDN1	Human	Hs_CLDN1_1_SG QuantiTect Primer Assay	QT00225764
CDLN2	Human	FW<br>REV	CGGGACTTCTACTCACCACTG<br>GGATGATTCCAGCTATCAGGGA
CLDN3	Human	Hs_CLDN3_1_SG QuantiTect Primer Assay	QT00201376
CLDN4	Human	Hs_CLDN4_1_SG QuantiTect Primer Assay	QT00241073
CLDN7	Human	Hs_CLDN7_1_SG QuantiTect Primer Assay	QT00236061
CRB3	Human	FW<br>REV	CCTTCATCCACCAGCTCCAG<br>GCCAAGAGGGAGAAGACCAC
GAPDH	Human	Hs_GAPDH_1_SG QuantiTect Primer Assay	QT00079247
IL10RB	Human	FW<br>REV	ATGAGCATTCAGACTGGGTAAAC<br>TTTTAGGGGCTAAGAAACGCAT
IL22	Human	FW<br>REV	GCTTGACAAGTCCAACTTCCA<br>GCTCACTCATACTGACTCCGT
IL22RA1	Human	FW<br>REV	CCGGCTAACCCTGGAAGAC<br>TCCAAGGTGCATTTGGTAGGT
JAK1	Human	FW<br>REV	CCACTACCGGATGAGGTTCTA<br>GGGTCTCGAATAGGAGCCAG
JAM2	Human	Hs_JAM2_1_SG QuantiTect Primer Assay	QT00078764
MAPK1	Human	FW<br>REV	TACACCAACCTCTCGTACATCG<br>CATGTCTGAAGCGCAGTAAGATT
MAPK9	Human	FW<br>REV	GAAACTAAGCCGTCCTTTTCAGA<br>TCCAGCTCCATGTGAATAACCT
MUC1	Human	Hs_MUC1_1_SG QuantiTect Primer Assay	QT00015379
MUC13	Human	Hs_MUC13_1_SG QuantiTect Primer Assay	QT00002478

PRKCZ	Human	FW<br>REV	ATGACGAGGA-TATTGACTGGGT<br>CAGGAGTGTAATCCGAC-CAGG
ROCK2	Human	FW<br>REV	TGGTTTCTATGGGCGA-GAATGT<br>CAAGTCGTAC-CTCCCTATCTGTT
SCRIB	Human	FW<br>REV	CCTCTGTCAAGGGAG-TGTCG<br>CCCGAGAGATGAA-TATGCCCTC
SNAI1	Human	FW<br>REV	CACTATGCCGCGCTCTTTC<br>GGTCGTAGGGCTGCTG-GAA
TJP2	Human	Hs_TJP2_1_SG QuantiTect Primer Assay	QT00010290
TLR4	Human	Hs_TLR4_1_SG QuantiTect Primer Assay	QT00035238
ZEB1	Human	FW<br>REV	TTACACCTTTGCATA-CAGAACCC<br>TTTACGAT-TACACCCAGACTGC
Ace2	Mouse	FW<br>REV	TGGTCTTCTGCCATCCGATT<br>CCATCCACCTCCAC-TTCTCTAA
Cdh1	Mouse	FW<br>REV	CAGTTCCGAGGTCTACAC-CTT<br>TGAATCGGGAG-TCTTCCGAAAA
Ceacam1	Mouse	FW<br>REV	TCACGGGGCAAGCATA-CAG<br>TCGCCTGAGTACGACGA-TAGT
Ceacam5	Mouse	FW<br>REV	TAACCATTGAATT-AGAGCCACCC<br>GGACCCAGCACAG-TAACTTTAGT
Cldn1	Mouse	FW<br>REV	TGCCCCAGTGGAAGAT-TTACT<br>CTTTGCGAAACGCAG-GACAT
Cldn2	Mouse	FW<br>REV	CAACTGGTGGGC-TACATCCTA<br>CCCTTGGAAAA-GCCAACCC
Cldn3	Mouse	FW<br>REV	ACCAACTGCGTACAA-GACGAG<br>CGGGCACCAAC-GGGTTATAG
Cldn4	Mouse	FW<br>REV	ATGGCGTCTATGGGAC-TACAG<br>GAGCGCACAACTCAG-GATG
Cldn7	Mouse	FW<br>REV	GGCCTGATAGCGAG-CACTG<br>TGGCGACAAACATGGCTAAGA

Crb3	Mouse	FW<br>REV	CACCG-GACCCTTTCACAAATA<br>CCCACTGCTATAAGGAG-GACT
Il10rb	Mouse	FW<br>REV	ACCTGCTTTCCCCAAAAC-GAA<br>TGAGAGAAGTCGCAC-TGAGTC
Il22ra	Mouse	FW<br>REV	ATGAAGACACTACTGAC-CATCCT<br>CAGCCAC-TTTCTCTCTCCGT
Jak1	Mouse	FW<br>REV	CTCTCTGTCACAAC-CTCTTCGC<br>TTGGTAAAGTAGAAC-CTCATGCG
Jam2	Mouse	FW<br>REV	GTGCCCAC-TTCTGTTATGACTG<br>TTCCCTAGCAAACTT-GTGCCA
Mapk1	Mouse	FW<br>REV	GGTT-GTTCCCAAATGCTGACT<br>CAACTTCAATCCTCTT-GTGAGGG
Mapk9	Mouse	FW<br>REV	AGTGATTGATCCAGACAA-GCG<br>GCGGGGTCAT-ACCAAACAGTA
Muc1	Mouse	FW<br>REV	GGTTGCTTTGGC-TATCGTCTATTT<br>AAA-GATGTCCAGCTGCCCATA
Muc2	Mouse	FW<br>REV	ATGCCCACCTCCTCAAA-GAC<br>GTAGTTTCCGTT-GGAACAGTGAA
Muc13	Mouse	FW<br>REV	GCCAGTCCTCCCACCAC-GGTA<br>CTGGGAC-CTGTGCTTCCACCG
aPkcz	Mouse	FW<br>REV	GCGTG-GATGCCATGACAACAT<br>GGCTCTTGG-GAAGGCATGACA
Rock2	Mouse	FW<br>REV	TTGGTTCGTCATAAGGCATCAC<br>TGTTGGCAAAGGCCATAA-TATCT
Rpl4	Mouse	FW<br>REV	CCGTCCCCTCATA-TCGGTGTA<br>GCATAGGGCTGTCTGTT-GTTTTT
Scrib	Mouse	FW<br>REV	CCTGGGCATCAGTATCG-CAG<br>GCCCTCGTCATCTCCTTTGT
Snai1	Mouse	FW<br>REV	ATCTCTTCACATCCGAG-TGG

			GAA-GATGCACATCCGAAGC
Tjp2	Mouse	FW<br>REV	ATGGGAGCAGTACAC-CGTGA<br>TGAC-CACCCTGTCATTTTCTTG
Tlr4	Mouse	FW<br>REV	ATGGCATGGCTTACAC-CACC<br>GAGGCCAATTTT-GTCTCCACA
Zeb1	Mouse	FW<br>REV	GCTGGCAAGACAAC-GTGAAAG<br>GCCTCAGGATAAATGAC-GGC

Table S2. Gene selection based on their involvement in intestinal barrier function.

Class	Gene	Description
Tight junction	CGN	Cingulin
Tight junction	CGNL1	Cingulin Like 1
Tight junction	CLDN1	Claudin 1
Tight junction	CLDN2	Claudin 2
Tight junction	CLDN3	Claudin 3
Tight junction	CLDN4	Claudin 4
Tight junction	CLDN7	Claudin 7
Tight junction	CLDN12	Claudin 12
Tight junction	CLDN14	Claudin 14
Tight junction	CLDN15	Claudin 15
Tight junction	CLDN16	Claudin 16
Tight junction	CLDN18	Claudin 18
Tight junction	JAM2	Junctional adhesion molecule 2
Tight junction	JAM3	Junctional adhesion molecule 3
Tight junction	JAM4/IGSF5	Junctional adhesion molecule 4
Tight junction	JAMA/F11R	Junctional adhesion molecule A
Tight junction	MARVELD1	MARVEL Domain Containing 1
Tight junction	MARVELD2	MARVEL Domain Containing 2
Tight junction	MARVELD3	MARVEL Domain Containing 3
Tight junction	OCLN	Occludin
Tight junction	TJP1	Tight junction protein 1
Tight junction	TJP2	Tight junction protein 2
Tight junction	TJP3	Tight junction protein 3
Adherens junctions	CDH1	E-cadherin
Adherens junctions	CTNNA1	Catenin A1
Adherens junctions	CTNNB1	Catenin B1
Adherens junctions	CTNND1	Catenin D1
Adherens junctions	VIM	Vimentin
Desmosomes	DSC1	Desmocollin 1
Desmosomes	DSC2	Desmocollin 2
Desmosomes	DSC3	Desmocollin 3
Desmosomes	DSG1	Desmoglein 1
Desmosomes	DSG2	Desmoglein 2
Desmosomes	DSG3	Desmoglein 3
Desmosomes	DSG4	Desmoglein 4
Desmosomes	DSP	Desmoplakin
Desmosomes	JUP	Junction Plakoglobin
Desmosomes	PKP1	Plakophilin 1
Desmosomes	PKP2	Plakophilin 2

Desmosomes	PKP3	Plakophilin 3
Desmosomes	PKP4	Plakophilin 4
Desmosomes	PPL	Periplakin
Hemidesmosomes	DST	Dystonin
Hemidesmosomes	ITGA6	Integrin Subunit Alpha64
Hemidesmosomes	ITGB4	Integrin Subunit Beta 4
Hemidesmosomes	PLEC	Plectin
Cytoskeleton	MYL2	Myosin Light Chain 2
Cytoskeleton	MYL5	Myosin Light Chain 5
Cytoskeleton	MYL6	Myosin Light Chain 6
Cytoskeleton	MYL6B	Myosin Light Chain 6B
Cytoskeleton	MYL7	Myosin Light Chain 7
Cytoskeleton	MYL9	Myosin Light Chain 9
Cytoskeleton	MYL12A	Myosin Light Chain 12A
Cytoskeleton	MYL12B	Myosin Light Chain 12B
Cytoskeleton	MYLK	Myosin Light Chain Kinase
Cytoskeleton	MYO9B	Myosin IXB
Mucus layer	MUC1	Mucin 1
Mucus layer	MUC2	Mucin 2
Mucus layer	MUC3A	Mucin 3A
Mucus layer	MUC12	Mucin 12
Mucus layer	MUC13	Mucin 13
Mucus layer	MUC17	Mucin 17
Mucus layer	MUC20	Mucin 20
Mucus layer	TFF1	Trefoil Factor 1
Mucus layer	TFF2	Trefoil Factor 2
Mucus layer	TFF3	Trefoil Factor 3
Epithelial immunity	ACE2	Angiotensin Converting Enzyme 2
Epithelial immunity	CEACAM1	CEA Cell Adhesion Molecule 1
Epithelial immunity	CEACAM18	CEA Cell Adhesion Molecule 18
Epithelial immunity	CEACAM19	CEA Cell Adhesion Molecule 19
Epithelial immunity	CEACAM5	CEA Cell Adhesion Molecule 5
Epithelial immunity	CEACAM6	CEA Cell Adhesion Molecule 6
Epithelial immunity	CEACAM8	CEA Cell Adhesion Molecule 8
Epithelial immunity	EPCAM	Epithelial Cell Adhesion Molecule
Epithelial immunity	TLR3	Toll Like Receptor 3
Epithelial immunity	TLR4	Toll Like Receptor 4
Epithelial immunity	TLR5	Toll Like Receptor 5
Epithelial immunity	TLR6	Toll Like Receptor 6
Cell polarity	CRB3	Crumbs Cell Polarity Complex Component 3
Cell polarity	DLG1	Discs Large MAGUK Scaffold Protein 1
Cell polarity	DLG2	Discs Large MAGUK Scaffold Protein 2
Cell polarity	DLG3	Discs Large MAGUK Scaffold Protein 3
Cell polarity	DLG4	Discs Large MAGUK Scaffold Protein 4
Cell polarity	DLG5	Discs Large MAGUK Scaffold Protein 5
Cell polarity	LLGL2	LLGL Scribble Cell Polarity Complex Component 2
Cell polarity	MPP5/PALS1	Protein Associated With LIN7 1, MAGUK P55 Family Member
Cell polarity	PARD3A	Par-3 Family Cell Polarity Regulator Alpha
Cell polarity	PARD3B	Par-3 Family Cell Polarity Regulator Beta
Cell polarity	PARD6A	Par-6 Family Cell Polarity Regulator Alpha
Cell polarity	PARD6B	Par-6 Family Cell Polarity Regulator Beta
Cell polarity	PATJ	PATJ Crumbs Cell Polarity Complex Component
Cell polarity	PRKCA	Protein Kinase C Alpha
Cell polarity	PRKCD	Protein Kinase C Delta
Cell polarity	PRKCE	Protein Kinase C Epsilon
Cell polarity	PRKCI	Protein Kinase C Iota
Cell polarity	PRKCZ	Protein Kinase C Zeta

Cell polarity	PRKD3	Protein Kinase D3
Cell polarity	PRKDC	Protein Kinase D Gamma
Cell polarity	SCRIB	Scribble Planar Cell Polarity Protein
Regulating proteins	AFDN	Afadin, Adherens Junction Formation Factor
Regulating proteins	AKT1	AKT Serine/Threonine Kinase 1
Regulating proteins	AMOT	Angiomotin
Regulating proteins	AMOTL1	Angiomotin Like 1
Regulating proteins	AMOTL2	Angiomotin Like 2
Regulating proteins	ARHGAP17	Rho GTPase Activating Protein 17
Regulating proteins	ARHGAP21	Rho GTPase Activating Protein 21
Regulating proteins	ARNT	Aryl Hydrocarbon Receptor Nuclear Translocator
Regulating proteins	CBL	Cbl Proto-Oncogene
Regulating proteins	CDC42	Cell Division Cycle 42
Regulating proteins	CDX1	Caudal Type Homeobox 1
Regulating proteins	CDX2	Caudal Type Homeobox 2
Regulating proteins	CEBPA	CCAAT Enhancer Binding Protein Alpha
Regulating proteins	CEBPB	CCAAT Enhancer Binding Protein Beta
Regulating proteins	CXADR	CXADR Ig-Like Cell Adhesion Molecule
Regulating proteins	EGFR	Epidermal Growth Factor Receptor
Regulating proteins	EHF	ETS Homologous Factor
Regulating proteins	ERBB2	Erb-B2 Receptor Tyrosine Kinase 2
Regulating proteins	ERBB3	Erb-B2 Receptor Tyrosine Kinase 3
Regulating proteins	ERK1/MAPK3	Mitogen-Activated Protein Kinase 3
Regulating proteins	ERK2/MAPK1	Mitogen-Activated Protein Kinase 1
Regulating proteins	EZH2	Enhancer Of Zeste 2 Polycomb Repressive Complex 2 Subunit
Regulating proteins	F2RL2	Coagulation Factor II Thrombin Receptor Like 2
Regulating proteins	FAK/PTK2	Protein Tyrosine Kinase 2
Regulating proteins	FOXO4	Forkhead Box O4
Regulating proteins	GNA12	G Protein Subunit Alpha 12
Regulating proteins	HDAC2	Histone Deacetylase 2
Regulating proteins	HIF1A	Hypoxia Inducible Factor 1 Subunti Alpha
Regulating proteins	HNF1A	HNF1 Homeobox A
Regulating proteins	HNF4A	Hepatocyte Nuclear Factor 4 Alpha
Regulating proteins	JAK1	Janus Kinase 1
Regulating proteins	JAK2	Janus Kinase 2
Regulating proteins	JAK3	Janus Kinase 3
Regulating proteins	JNK1/MAPK8	Mitogen-Activated Protein Kinase 8
Regulating proteins	JNK2/MAPK9	Mitogen-Activated Protein Kinase 9
Regulating proteins	MAGI1	Membrane Associated Guanylate Kinase, WW And PDZ Domain Containing 1
Regulating proteins	MAGI2	Membrane Associated Guanylate Kinase, WW And PDZ Domain Containing 2
Regulating proteins	MAGI3	Membrane Associated Guanylate Kinase, WW And PDZ Domain Containing 3
Regulating proteins	MAPK13	Mitogen-Activated Protein Kinase 13
Regulating proteins	MEP1A	Meprin A Subunit Alpha
Regulating proteins	MYC	MYC Proto-Oncogene, BHLH Transcription Factor
Regulating proteins	NFKB1	Nuclear Factor Kappa B1
Regulating proteins	NFKBIA	Nuclear Factor Kappa B1 Inhibitor Alpha
Regulating proteins	PTGER4	Prostaglandin E Receptor 4
Regulating proteins	PTPN2	Protein Tyrosine Phosphatase Non-Receptor Type 2
Regulating proteins	RAC1	Rac Family Small GTPase 1
Regulating proteins	RAC2	Rac Family Small GTPase 2
Regulating proteins	RAC3	Rac Family Small GTPase 3
Regulating proteins	RETNLB	Resistin Like Beta
Regulating proteins	RHOA	Ras Homolog Family Member A

Regulating proteins	ROCK1	Rho Associated Coiled-Coil Containing Protein Kinase 1
Regulating proteins	ROCK2	Rho Associated Coiled-Coil Containing Protein Kinase 2
Regulating proteins	SH3KBP1/CIN85	SH3 Domain Containing Kinase Binding Protein 1
Regulating proteins	SMARCA4	SWI/SNF Related, Matrix Associated, Actin Dependent Regulator Of Chromatin, Subfamily A, Member 4
Regulating proteins	SMURF1	SMAD Specific E3 Ubiquitin Protein Ligase 1
Regulating proteins	SMURF2	SMAD Specific E3 Ubiquitin Protein Ligase 2
Regulating proteins	SNAI1	Snail Family Transcriptional Repressor 1
Regulating proteins	SPATA13	Spermatogenesis Associated 13
Regulating proteins	SPINT2	Serine Peptidase Inhibitor, Kunitz Type 2
Regulating proteins	STAT3	Signal Transducer And Activator Of Transcription 3
Regulating proteins	STAT5A	Signal Transducer And Activator Of Transcription 5 Alpha
Regulating proteins	STAT5B	Signal Transducer And Activator Of Transcription 5 Beta
Regulating proteins	SYMPK	Symplekin Scaffold Protein
Regulating proteins	TCF4	Transcription Factor 4
Regulating proteins	VDR	Vitamin D Receptor
Regulating proteins	WAS	WASP Actin Nucleation Promoting Factor
Regulating proteins	WASF1	WASP Family Member 1
Regulating proteins	WNT10A	Wnt Family Member 10A
Regulating proteins	WNT10B	Wnt Family Member 10B
Regulating proteins	WNT11	Wnt Family Member 11
Regulating proteins	WNT2B	Wnt Family Member 2B
Regulating proteins	WNT4	Wnt Family Member 4
Regulating proteins	WNT9A	Wnt Family Member 9A
Regulating proteins	ZEB1	Zinc Finger E-Box Binding Homeobox 1

Adapted from Vancamelbeke et al. (2017) [1].

References

1. Vancamelbeke, M.; Vanuytsel, T.; Farré, R.; Verstockt, S.; Ferrante, M.; Van Assche, G.; Rutgeerts, P.; Schuit, F.; Vermeire, S.; Arijs, I.; et al. Genetic and Transcriptomic Bases of Intestinal Epithelial Barrier Dysfunction in Inflammatory Bowel Disease. *Inflamm. Bowel. Dis*. **2017**, *23*, 1718–1729.
