# Supplementary material for: IL-22-Activated MUC13 Impacts on Colonic Barrier Function through JAK1/STAT3, SNAI1/ZEB1 and ROCK2/MAPK Signaling
Source: Cells. 2023 Apr 23;12(9):1224. doi: 10.3390/cells12091224 (PMC10177587; doi:10.3390/cells12091224)
Supplement: Supplementary file 1 [file cells-12-01224-s001.zip › cells-2275517-supplementary_proofreading completed.docx]

Supplementary Material


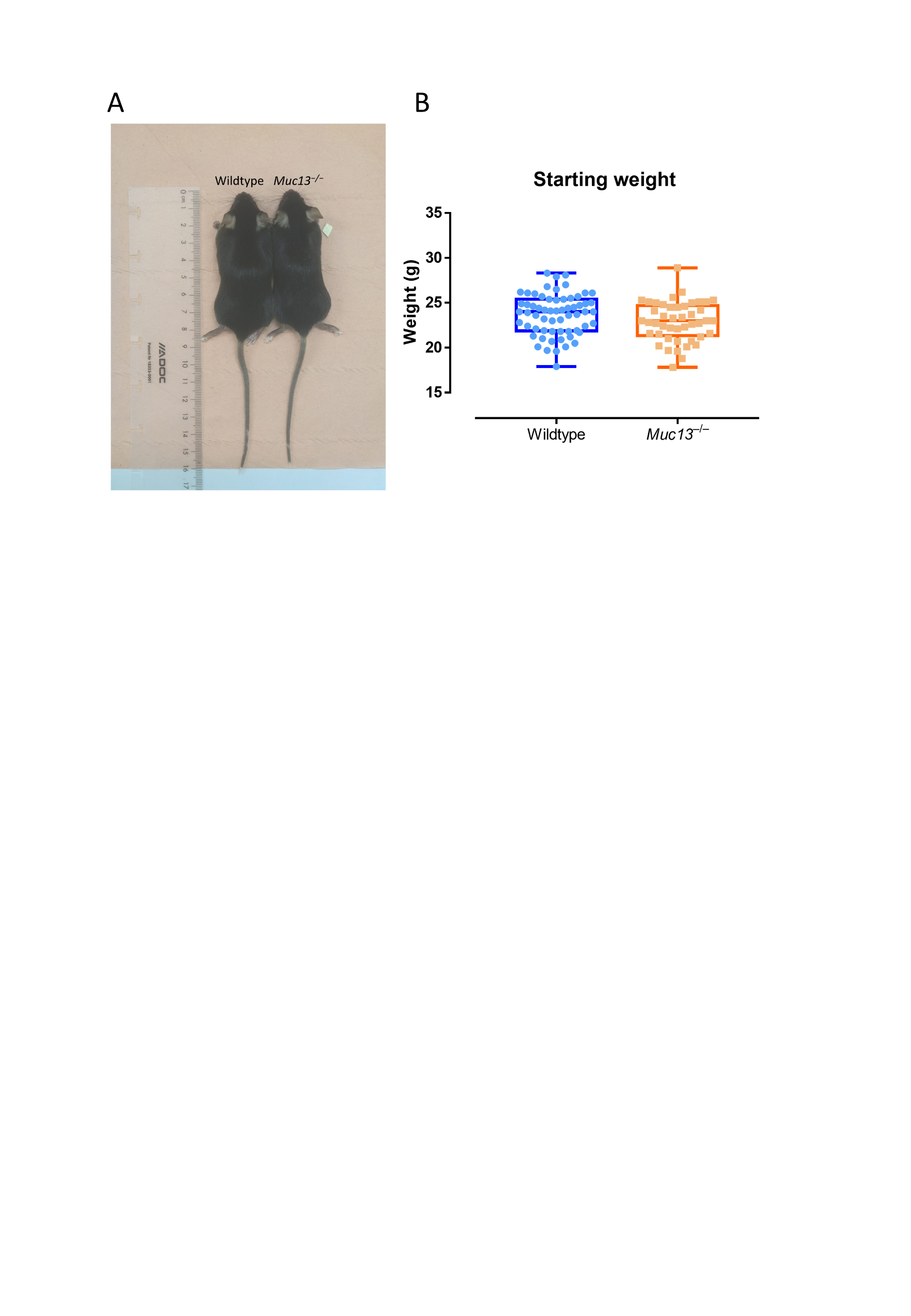


**Figure S1.** Comparison of healthy wildtype and *Muc13*^−/−^ mice. **(A)** Representative photo of 7-week old male wildtype and *Muc13*^−/−^ mice. **(B)** Weight of wildtype and *Muc13*^−/−^ mice at the start of the DSS experiments.


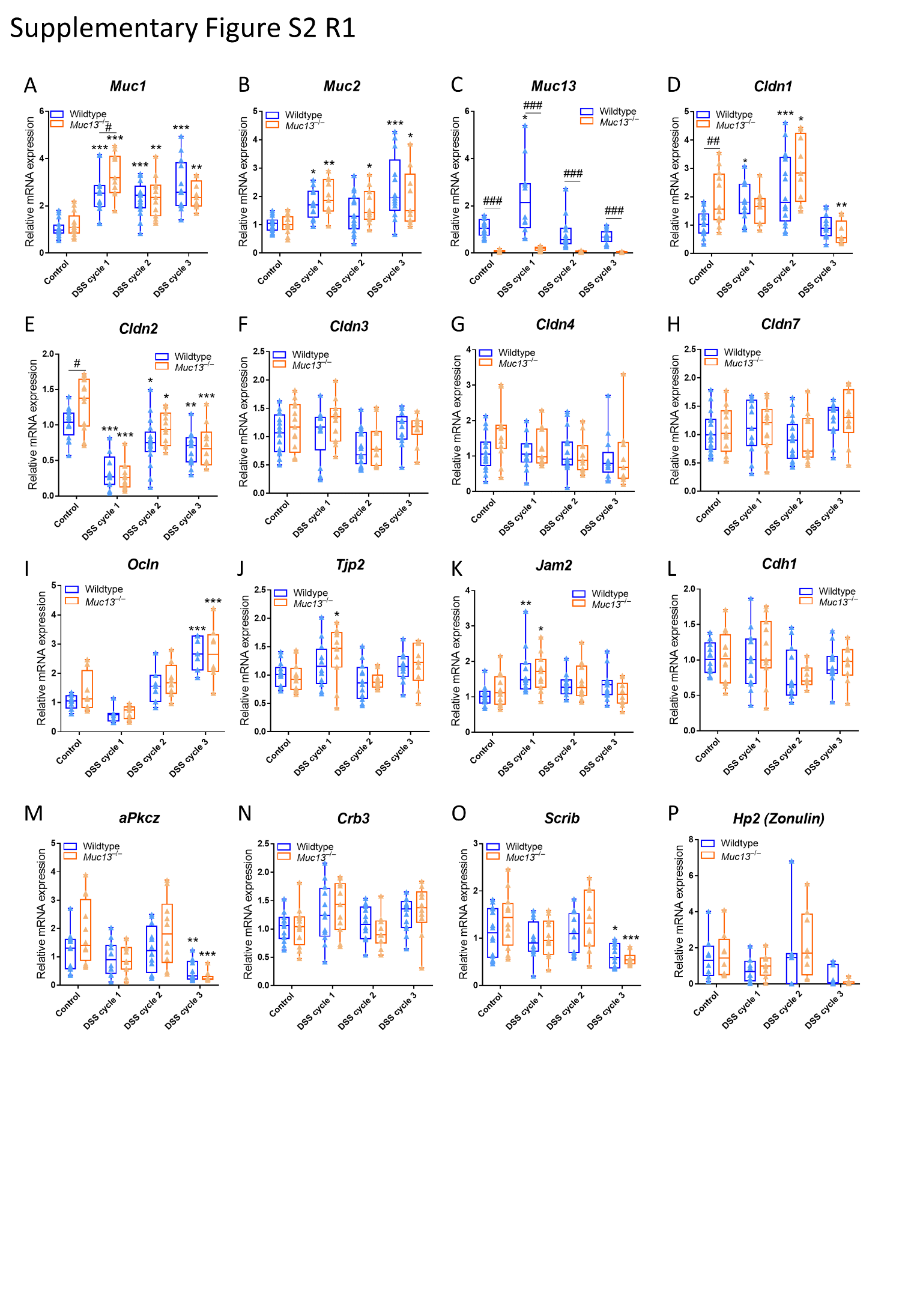


**Figure S2.** Gene expression of mucins, intercellular junctions and cell polarity complexes in the colon of wildtype and *Muc13*^−/−^ mice during the course of DSS-colitis. Relative mRNA expression of (A-C) mucins (*Muc1*, *Muc2*, *Muc13*), (D-K) tight junctions (*Cldn1*, *Cldn2*, *Cldn3*, *Cldn4*, *Cldn7*, *Ocln*, *Tjp2*, *Jam2*), (L) adherens junctions (*Cdh1*), and (M-O) cell polarity subunits (*Scrib*, *Crb3*, *aPKcz*) and *Hp2* (Zonulin) in the colon of healthy and DSS-colitis wildtype and *Muc13*^−/−^ mice (N=7-17/group/gene). Significant differences between control and colitis mice are indicated by *p<0.05; **p<0.01; ***p<0.001 and between wildtype (WT) and *Muc13*^−/−^ by #p<0.05; ##p<0.01; ###p<0.001 (Two-Way ANOVA).


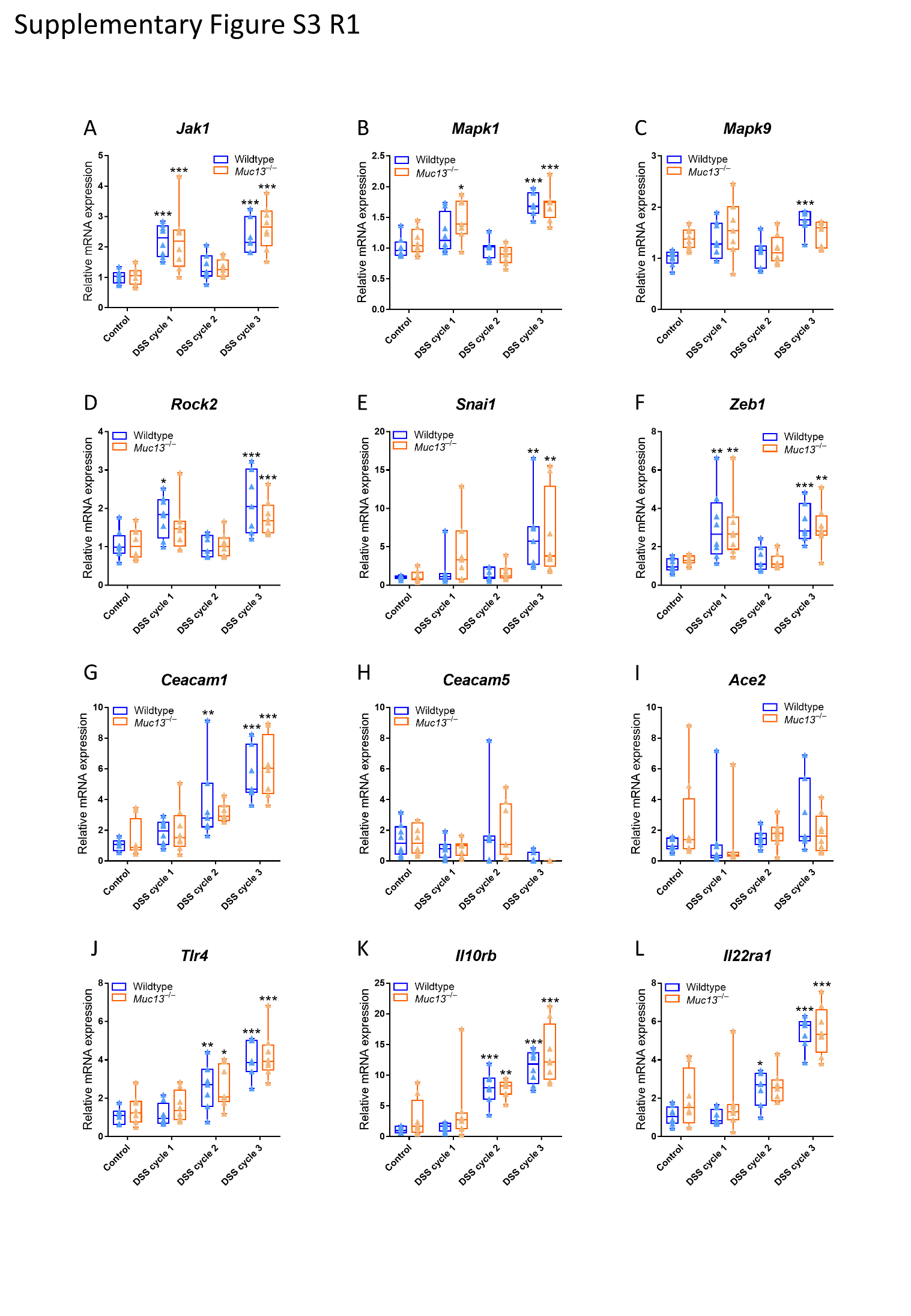


**Figure S3.** mRNA expression analysis of major regulators and epithelial immunity mediators involved in intestinal barrier homeostasis in the colon of wildtype and *Muc13*^−/−^ mice during the course of DSS-colitis. Relative mRNA expression of (**A**–**F**) major regulators (*Jak1*, *Mapk1*, *Mapk9*, *Rock2*, *Snai1*, *Zeb1*) and (G-L) epithelial immunity mediators (*Ceacam1*, *Ceacam5*, *Ace2*, *Tlr4*, *Il10rb*, *Il22ra*) (N=7-17/group/gene). Significant differences between control and colitis mice are indicated by *p<0.05; **p<0.01; ***p<0.001 (Two-Way ANOVA).

**Table S1.** Primer sequences used in qPCR assays.

| Gene name | Species | Primer | Primer sequence (5’–3’) |
| --- | --- | --- | --- |
| ACE2 | Human | Hs_ACE2_1_SG QuantiTect Primer Assay | QT00034055 |
| ACTB | Human | Hs_ACTB_1_SG QuantiTect Primer Assay | QT00095431 |
| CEACAM1 | Human | Hs_CEACAM1_1_SG QuantiTect Primer Assay | QT00072429 |
| CEACAM5 | Human | Hs_CEACAM5_1_SG QuantiTect Primer Assay | QT00032991 |
| CEACAM6 | Human | Hs_CEACAM6_1_SG QuantiTect Primer Assay | QT00066521 |
| CDH1 | Human | Hs_CDH1_1_SG QuantiTect Primer Assay | QT00080143 |
| CLDN1 | Human | Hs_CLDN1_1_SG QuantiTect Primer Assay | QT00225764 |
| CDLN2 | Human | FW  REV | CGGGACTTCTACTCACCACTG  GGATGATTCCAGCTATCAGGGA |
| CLDN3 | Human | Hs_CLDN3_1_SG QuantiTect Primer Assay | QT00201376 |
| CLDN4 | Human | Hs_CLDN4_1_SG QuantiTect Primer Assay | QT00241073 |
| CLDN7 | Human | Hs_CLDN7_1_SG QuantiTect Primer Assay | QT00236061 |
| CRB3 | Human | FW  REV | CCTTCATCCACCAGCTCCAG  GCCAAGAGGGAGAAGACCAC |
| GAPDH | Human | Hs_GAPDH_1_SG QuantiTect Primer Assay | QT00079247 |
| IL10RB | Human | FW  REV | ATGAGCATTCAGACTGGGTAAAC  TTTTAGGGGCTAAGAAACGCAT |
| IL22 | Human | FW  REV | GCTTGACAAGTCCAACTTCCA  GCTCACTCATACTGACTCCGT |
| IL22RA1 | Human | FW  REV | CCGGCTAACCCTGGAAGAC  TCCAAGGTGCATTTGGTAGGT |
| JAK1 | Human | FW  REV | CCACTACCGGATGAGGTTCTA  GGGTCTCGAATAGGAGCCAG |
| JAM2 | Human | Hs_JAM2_1_SG QuantiTect Primer Assay | QT00078764 |
| MAPK1 | Human | FW  REV | TACACCAACCTCTCGTACATCG  CATGTCTGAAGCGCAGTAAGATT |
| MAPK9 | Human | FW  REV | GAAACTAAGCCGTCCTTTTCAGA TCCAGCTCCATGTGAATAACCT |
| MUC1 | Human | Hs_MUC1_1_SG QuantiTect Primer Assay | QT00015379 |
| MUC13 | Human | Hs_MUC13_1_SG QuantiTect Primer Assay | QT00002478 |
| PRKCZ | Human | FW  REV | ATGACGAGGATATTGACTGGGT  CAGGAGTGTAATCCGACCAGG |
| ROCK2 | Human | FW  REV | TGGTTTCTATGGGCGAGAATGT  CAAGTCGTACCTCCCTATCTGTT |
| SCRIB | Human | FW  REV | CCTCTGTCAAGGGAGTGTCG  CCCGAGAGATGAATATGCCCTC |
| SNAI1 | Human | FW  REV | CACTATGCCGCGCTCTTTC  GGTCGTAGGGCTGCTGGAA |
| TJP2 | Human | Hs_TJP2_1_SG QuantiTect Primer Assay | QT00010290 |
| TLR4 | Human | Hs_TLR4_1_SG QuantiTect Primer Assay | QT00035238 |
| ZEB1 | Human | FW  REV | TTACACCTTTGCATACAGAACCC  TTTACGATTACACCCAGACTGC |
| Ace2 | Mouse | FW  REV | TGGTCTTCTGCCATCCGATT  CCATCCACCTCCACTTCTCTAA |
| Cdh1 | Mouse | FW  REV | CAGTTCCGAGGTCTACACCTT  TGAATCGGGAGTCTTCCGAAAA |
| Ceacam1 | Mouse | FW  REV | TCACGGGGCAAGCATACAG  TCGCCTGAGTACGACGATAGT |
| Ceacam5 | Mouse | FW  REV | TAACCATTGAATTAGAGCCACCC  GGACCCAGCACAGTAACTTTAGT |
| Cldn1 | Mouse | FW  REV | TGCCCCAGTGGAAGATTTACT  CTTTGCGAAACGCAGGACAT |
| Cldn2 | Mouse | FW  REV | CAACTGGTGGGCTACATCCTA  CCCTTGGAAAAGCCAACCC |
| Cldn3 | Mouse | FW  REV | ACCAACTGCGTACAAGACGAG  CGGGCACCAACGGGTTATAG |
| Cldn4 | Mouse | FW  REV | ATGGCGTCTATGGGACTACAG  GAGCGCACAACTCAGGATG |
| Cldn7 | Mouse | FW  REV | GGCCTGATAGCGAGCACTG  TGGCGACAAACATGGCTAAGA |
| Crb3 | Mouse | FW  REV | CACCGGACCCTTTCACAAATA  CCCACTGCTATAAGGAGGACT |
| Il10rb | Mouse | FW  REV | ACCTGCTTTCCCCAAAACGAA  TGAGAGAAGTCGCACTGAGTC |
| Il22ra | Mouse | FW  REV | ATGAAGACACTACTGACCATCCT  CAGCCACTTTCTCTCTCCGT |
| Jak1 | Mouse | FW  REV | CTCTCTGTCACAACCTCTTCGC  TTGGTAAAGTAGAACCTCATGCG |
| Jam2 | Mouse | FW  REV | GTGCCCACTTCTGTTATGACTG  TTCCCTAGCAAACTTGTGCCA |
| Mapk1 | Mouse | FW  REV | GGTTGTTCCCAAATGCTGACT  CAACTTCAATCCTCTTGTGAGGG |
| Mapk9 | Mouse | FW  REV | AGTGATTGATCCAGACAAGCG  GCGGGGTCATACCAAACAGTA |
| Muc1 | Mouse | FW  REV | GGTTGCTTTGGCTATCGTCTATTT  AAAGATGTCCAGCTGCCCATA |
| Muc2 | Mouse | FW  REV | ATGCCCACCTCCTCAAAGAC  GTAGTTTCCGTTGGAACAGTGAA |
| Muc13 | Mouse | FW  REV | GCCAGTCCTCCCACCACGGTA  CTGGGACCTGTGCTTCCACCG |
| aPkcz | Mouse | FW  REV | GCGTGGATGCCATGACAACAT  GGCTCTTGGGAAGGCATGACA |
| Rock2 | Mouse | FW  REV | TTGGTTCGTCATAAGGCATCAC  TGTTGGCAAAGGCCATAATATCT |
| Rpl4 | Mouse | FW  REV | CCGTCCCCTCATATCGGTGTA  GCATAGGGCTGTCTGTTGTTTTT |
| Scrib | Mouse | FW  REV | CCTGGGCATCAGTATCGCAG  GCCCTCGTCATCTCCTTTGT |
| Snai1 | Mouse | FW  REV | ATCTCTTCACATCCGAGTGG  GAAGATGCACATCCGAAGC |
| Tjp2 | Mouse | FW  REV | ATGGGAGCAGTACACCGTGA  TGACCACCCTGTCATTTTCTTG |
| Tlr4 | Mouse | FW  REV | ATGGCATGGCTTACACCACC  GAGGCCAATTTTGTCTCCACA |
| Zeb1 | Mouse | FW  REV | GCTGGCAAGACAACGTGAAAG  GCCTCAGGATAAATGACGGC |

**Table S2.** Gene selection based on their involvement in intestinal barrier function.

| Class | Gene | Description |
| --- | --- | --- |
| Tight junction | CGN | Cingulin |
| Tight junction | CGNL1 | Cingulin Like 1 |
| Tight junction | CLDN1 | Claudin 1 |
| Tight junction | CLDN2 | Claudin 2 |
| Tight junction | CLDN3 | Claudin 3 |
| Tight junction | CLDN4 | Claudin 4 |
| Tight junction | CLDN7 | Claudin 7 |
| Tight junction | CLDN12 | Claudin 12 |
| Tight junction | CLDN14 | Claudin 14 |
| Tight junction | CLDN15 | Claudin 15 |
| Tight junction | CLDN16 | Claudin 16 |
| Tight junction | CLDN18 | Claudin 18 |
| Tight junction | JAM2 | Junctional adhesion molecule 2 |
| Tight junction | JAM3 | Junctional adhesion molecule 3 |
| Tight junction | JAM4/IGSF5 | Junctional adhesion molecule 4 |
| Tight junction | JAMA/F11R | Junctional adhesion molecule A |
| Tight junction | MARVELD1 | MARVEL Domain Containing 1 |
| Tight junction | MARVELD2 | MARVEL Domain Containing 2 |
| Tight junction | MARVELD3 | MARVEL Domain Containing 3 |
| Tight junction | OCLN | Occludin |
| Tight junction | TJP1 | Tight junction protein 1 |
| Tight junction | TJP2 | Tight junction protein 2 |
| Tight junction | TJP3 | Tight junction protein 3 |
| Adherens junctions | CDH1 | E-cadherin |
| Adherens junctions | CTNNA1 | Catenin A1 |
| Adherens junctions | CTNNB1 | Catenin B1 |
| Adherens junctions | CTNND1 | Catenin D1 |
| Adherens junctions | VIM | Vimentin |
| Desmosomes | DSC1 | Desmocollin 1 |
| Desmosomes | DSC2 | Desmocollin 2 |
| Desmosomes | DSC3 | Desmocollin 3 |
| Desmosomes | DSG1 | Desmoglein 1 |
| Desmosomes | DSG2 | Desmoglein 2 |
| Desmosomes | DSG3 | Desmoglein 3 |
| Desmosomes | DSG4 | Desmoglein 4 |
| Desmosomes | DSP | Desmoplakin |
| Desmosomes | JUP | Junction Plakoglobin |
| Desmosomes | PKP1 | Plakophilin 1 |
| Desmosomes | PKP2 | Plakophilin 2 |
| Desmosomes | PKP3 | Plakophilin 3 |
| Desmosomes | PKP4 | Plakophilin 4 |
| Desmosomes | PPL | Periplakin |
| Hemidesmosomes | DST | Dystonin |
| Hemidesmosomes | ITGA6 | Integrin Subunit Alpha64 |
| Hemidesmosomes | ITGB4 | Integrin Subunit Beta 4 |
| Hemidesmosomes | PLEC | Plectin |
| Cytoskeleton | MYL2 | Myosin Light Chain 2 |
| Cytoskeleton | MYL5 | Myosin Light Chain 5 |
| Cytoskeleton | MYL6 | Myosin Light Chain 6 |
| Cytoskeleton | MYL6B | Myosin Light Chain 6B |
| Cytoskeleton | MYL7 | Myosin Light Chain 7 |
| Cytoskeleton | MYL9 | Myosin Light Chain 9 |
| Cytoskeleton | MYL12A | Myosin Light Chain 12A |
| Cytoskeleton | MYL12B | Myosin Light Chain 12B |
| Cytoskeleton | MYLK | Myosin Light Chain Kinase |
| Cytoskeleton | MYO9B | Myosin IXB |
| Mucus layer | MUC1 | Mucin 1 |
| Mucus layer | MUC2 | Mucin 2 |
| Mucus layer | MUC3A | Mucin 3A |
| Mucus layer | MUC12 | Mucin 12 |
| Mucus layer | MUC13 | Mucin 13 |
| Mucus layer | MUC17 | Mucin 17 |
| Mucus layer | MUC20 | Mucin 20 |
| Mucus layer | TFF1 | Trefoil Factor 1 |
| Mucus layer | TFF2 | Trefoil Factor 2 |
| Mucus layer | TFF3 | Trefoil Factor 3 |
| Epithelial immunity | ACE2 | Angiotensin Converting Enzyme 2 |
| Epithelial immunity | CEACAM1 | CEA Cell Adhesion Molecule 1 |
| Epithelial immunity | CEACAM18 | CEA Cell Adhesion Molecule 18 |
| Epithelial immunity | CEACAM19 | CEA Cell Adhesion Molecule 19 |
| Epithelial immunity | CEACAM5 | CEA Cell Adhesion Molecule 5 |
| Epithelial immunity | CEACAM6 | CEA Cell Adhesion Molecule 6 |
| Epithelial immunity | CEACAM8 | CEA Cell Adhesion Molecule 8 |
| Epithelial immunity | EPCAM | Epithelial Cell Adhesion Molecule |
| Epithelial immunity | TLR3 | Toll Like Receptor 3 |
| Epithelial immunity | TLR4 | Toll Like Receptor 4 |
| Epithelial immunity | TLR5 | Toll Like Receptor 5 |
| Epithelial immunity | TLR6 | Toll Like Receptor 6 |
| Cell polarity | CRB3 | Crumbs Cell Polarity Complex Component 3 |
| Cell polarity | DLG1 | Discs Large MAGUK Scaffold Protein 1 |
| Cell polarity | DLG2 | Discs Large MAGUK Scaffold Protein 2 |
| Cell polarity | DLG3 | Discs Large MAGUK Scaffold Protein 3 |
| Cell polarity | DLG4 | Discs Large MAGUK Scaffold Protein 4 |
| Cell polarity | DLG5 | Discs Large MAGUK Scaffold Protein 5 |
| Cell polarity | LLGL2 | LLGL Scribble Cell Polarity Complex Component 2 |
| Cell polarity | MPP5/PALS1 | Protein Associated With LIN7 1, MAGUK P55 Family Member |
| Cell polarity | PARD3A | Par-3 Family Cell Polarity Regulator Alpha |
| Cell polarity | PARD3B | Par-3 Family Cell Polarity Regulator Beta |
| Cell polarity | PARD6A | Par-6 Family Cell Polarity Regulator Alpha |
| Cell polarity | PARD6B | Par-6 Family Cell Polarity Regulator Beta |
| Cell polarity | PATJ | PATJ Crumbs Cell Polarity Complex Component |
| Cell polarity | PRKCA | Protein Kinase C Alpha |
| Cell polarity | PRKCD | Protein Kinase C Delta |
| Cell polarity | PRKCE | Protein Kinase C Epsilon |
| Cell polarity | PRKCI | Protein Kinase C Iota |
| Cell polarity | PRKCZ | Protein Kinase C Zeta |
| Cell polarity | PRKD3 | Protein Kinase D3 |
| Cell polarity | PRKDC | Protein Kinase D Gamma |
| Cell polarity | SCRIB | Scribble Planar Cell Polarity Protein |
| Regulating proteins | AFDN | Afadin, Adherens Junction Formation Factor |
| Regulating proteins | AKT1 | AKT Serine/Threonine Kinase 1 |
| Regulating proteins | AMOT | Angiomotin |
| Regulating proteins | AMOTL1 | Angiomotin Like 1 |
| Regulating proteins | AMOTL2 | Angiomotin Like 2 |
| Regulating proteins | ARHGAP17 | Rho GTPase Activating Protein 17 |
| Regulating proteins | ARHGAP21 | Rho GTPase Activating Protein 21 |
| Regulating proteins | ARNT | Aryl Hydrocarbon Receptor Nuclear Translocator |
| Regulating proteins | CBL | Cbl Proto-Oncogene |
| Regulating proteins | CDC42 | Cell Division Cycle 42 |
| Regulating proteins | CDX1 | Caudal Type Homeobox 1 |
| Regulating proteins | CDX2 | Caudal Type Homeobox 2 |
| Regulating proteins | CEBPA | CCAAT Enhancer Binding Protein Alpha |
| Regulating proteins | CEBPB | CCAAT Enhancer Binding Protein Beta |
| Regulating proteins | CXADR | CXADR Ig-Like Cell Adhesion Molecule |
| Regulating proteins | EGFR | Epidermal Growth Factor Receptor |
| Regulating proteins | EHF | ETS Homologous Factor |
| Regulating proteins | ERBB2 | Erb-B2 Receptor Tyrosine Kinase 2 |
| Regulating proteins | ERBB3 | Erb-B2 Receptor Tyrosine Kinase 3 |
| Regulating proteins | ERK1/MAPK3 | Mitogen-Activated Protein Kinase 3 |
| Regulating proteins | ERK2/MAPK1 | Mitogen-Activated Protein Kinase 1 |
| Regulating proteins | EZH2 | Enhancer Of Zeste 2 Polycomb Repressive Complex 2 Subunit |
| Regulating proteins | F2RL2 | Coagulation Factor II Thrombin Receptor Like 2 |
| Regulating proteins | FAK/PTK2 | Protein Tyrosine Kinase 2 |
| Regulating proteins | FOXO4 | Forkhead Box O4 |
| Regulating proteins | GNA12 | G Protein Subunit Alpha 12 |
| Regulating proteins | HDAC2 | Histone Deacetylase 2 |
| Regulating proteins | HIF1A | Hypoxia Inducible Factor 1 Subunti Alpha |
| Regulating proteins | HNF1A | HNF1 Homeobox A |
| Regulating proteins | HNF4A | Hepatocyte Nuclear Factor 4 Alpha |
| Regulating proteins | JAK1 | Janus Kinase 1 |
| Regulating proteins | JAK2 | Janus Kinase 2 |
| Regulating proteins | JAK3 | Janus Kinase 3 |
| Regulating proteins | JNK1/MAPK8 | Mitogen-Activated Protein Kinase 8 |
| Regulating proteins | JNK2/MAPK9 | Mitogen-Activated Protein Kinase 9 |
| Regulating proteins | MAGI1 | Membrane Associated Guanylate Kinase, WW And PDZ Domain Containing 1 |
| Regulating proteins | MAGI2 | Membrane Associated Guanylate Kinase, WW And PDZ Domain Containing 2 |
| Regulating proteins | MAGI3 | Membrane Associated Guanylate Kinase, WW And PDZ Domain Containing 3 |
| Regulating proteins | MAPK13 | Mitogen-Activated Protein Kinase 13 |
| Regulating proteins | MEP1A | Meprin A Subunit Alpha |
| Regulating proteins | MYC | MYC Proto-Oncogene, BHLH Transcription Factor |
| Regulating proteins | NFKB1 | Nuclear Factor Kappa B1 |
| Regulating proteins | NFKBIA | Nuclear Factor Kappa B1 Inhibitor Alpha |
| Regulating proteins | PTGER4 | Prostaglandin E Receptor 4 |
| Regulating proteins | PTPN2 | Protein Tyrosine Phosphatase Non-Receptor Type 2 |
| Regulating proteins | RAC1 | Rac Family Small GTPase 1 |
| Regulating proteins | RAC2 | Rac Family Small GTPase 2 |
| Regulating proteins | RAC3 | Rac Family Small GTPase 3 |
| Regulating proteins | RETNLB | Resistin Like Beta |
| Regulating proteins | RHOA | Ras Homolog Family Member A |
| Regulating proteins | ROCK1 | Rho Associated Coiled-Coil Containing Protein Kinase 1 |
| Regulating proteins | ROCK2 | Rho Associated Coiled-Coil Containing Protein Kinase 2 |
| Regulating proteins | SH3KBP1/CIN85 | SH3 Domain Containing Kinase Binding Protein 1 |
| Regulating proteins | SMARCA4 | SWI/SNF Related, Matrix Associated, Actin Dependent Regulator Of Chromatin, Subfamily A, Member 4 |
| Regulating proteins | SMURF1 | SMAD Specific E3 Ubiquitin Protein Ligase 1 |
| Regulating proteins | SMURF2 | SMAD Specific E3 Ubiquitin Protein Ligase 2 |
| Regulating proteins | SNAI1 | Snail Family Transcriptional Repressor 1 |
| Regulating proteins | SPATA13 | Spermatogenesis Associated 13 |
| Regulating proteins | SPINT2 | Serine Peptidase Inhibitor, Kunitz Type 2 |
| Regulating proteins | STAT3 | Signal Transducer And Activator Of Transcription 3 |
| Regulating proteins | STAT5A | Signal Transducer And Activator Of Transcription 5 Alpha |
| Regulating proteins | STAT5B | Signal Transducer And Activator Of Transcription 5 Beta |
| Regulating proteins | SYMPK | Symplekin Scaffold Protein |
| Regulating proteins | TCF4 | Transcription Factor 4 |
| Regulating proteins | VDR | Vitamin D Receptor |
| Regulating proteins | WAS | WASP Actin Nucleation Promoting Factor |
| Regulating proteins | WASF1 | WASP Family Member 1 |
| Regulating proteins | WNT10A | Wnt Family Member 10A |
| Regulating proteins | WNT10B | Wnt Family Member 10B |
| Regulating proteins | WNT11 | Wnt Family Member 11 |
| Regulating proteins | WNT2B | Wnt Family Member 2B |
| Regulating proteins | WNT4 | Wnt Family Member 4 |
| Regulating proteins | WNT9A | Wnt Family Member 9A |
| Regulating proteins | ZEB1 | Zinc Finger E-Box Binding Homeobox 1 |

Adapted from Vancamelbeke et al. (2017).

**References**

Vancamelbeke, M.; Vanuytsel, T.; Farré, R.; Verstockt, S.; Ferrante, M.; Van Assche, G.; Rutgeerts, P.; Schuit, F.; Vermeire, S.; Arijs, I.; et al. Genetic and Transcriptomic Bases of Intestinal Epithelial Barrier Dysfunction in Inflammatory Bowel Disease. *Inflamm. Bowel. Dis*. **2017**, *23*, 1718–1729.
